# Demonstration of the synergistic effect of biochar and *Trichoderma harzianum* on the development of *Ralstonia solanacearum* in eggplant

**DOI:** 10.3389/fmicb.2024.1360703

**Published:** 2024-04-25

**Authors:** Chaudhry Ali Ahmad, Adnan Akhter, Muhammad Saleem Haider, Muhammad Taqqi Abbas, Abeer Hashem, Graciela Dolores Avila-Quezada, Elsayed Fathi Abd_Allah

**Affiliations:** ^1^Department of Plant Pathology, Faculty of Agriculture Sciences, Quaid-e-Azam Campus, University of the Punjab, Lahore, Pakistan; ^2^Botany and Microbiology Department, College of Science, King Saud University, Riyadh, Saudi Arabia; ^3^Facultad de Ciencias Agrotecnológicas, Universidad Autónoma de Chihuahua, Chihuahua, Mexico; ^4^Plant Production Department, College of Food and Agricultural Sciences, King Saud University, Riyadh, Saudi Arabia

**Keywords:** carbon sequestration, plant protection, bacterial wilt, *Trichoderma harzianum*, soil amendment, organic agriculture

## Abstract

Soil degradation has been accelerated by the use of chemical pesticides and poor agricultural practices, which has had an impact on crop productivity. Recently, there has been a lot of interest in the use of eco-friendly biochar applications to enhance soil quality and sequester carbon in sustainable agriculture. This study aimed to determine the individual and combined effects of Leaf Waste Biochar (LWB) and the bio-control agent *Trichoderma harzianum* (BCA) on the development of bacterial wilt in eggplants (*Solanum melongena*) caused by *Ralstonia solanacearum* (RS). The effects of LWB and BCA on eggplant physiology and defense-related biochemistry were comprehensively examined. Inoculated (+RS) and un-inoculated (–RS) eggplants were grown in potting mixtures containing 3% and 6% (v/v) LWB, both with and without BCA. The percentage disease index was considerably reduced (90%) in plants grown in the 6% LWB+ BCA amended treatments. Moreover, the plants grown in LWB and inoculated with BCA had higher phenolics, flavonoids and peroxidase contents compared to the non-amended control. The level of NPK was significantly increased (92.74% N, 76.47% P, 53.73% K) in the eggplants cultivated in the 6% LWB + BCA composition. This study has shown that the association of *T. harzianum* with biochar improved plant growth and reduced *R. solanacearum* induced wilt. Furthermore, the combined impact of biochar and *T. harzianum* was greater in terms of wilt suppression and increase in plant physiological measurements when the biochar concentration was 6%. Biochar and bio-control agents triggered biochemical alterations, thus enhancing the management of disease-infested soils.

## 1 Introduction

Although global food production has increased dramatically in recent decades, approximately one billion people still suffer from malnutrition (Semida et al., 2019). There is a growing global interest in decreasing food losses and wastage to improve food security (Alexander et al., 2017). Plant diseases, a major cause of crop losses, pose a significant risk to global food security and account for 10 to 16% of annual crop losses (Bag et al., 2017). Phyto-pathogenic diseases continue to threaten Pakistan's economy, causing billions of dollars in losses (Ali et al., 2018).

*Solanum melongena* L., commonly known as eggplant, brinjal, and aubergine, belongs to the Solanaceae family. This family renowned for its nutritional advantages due to the rich presence of bioactive substances, including vitamins, minerals, phenolics, dry matter content, and macronutrients. The primary source of eggplant's health benefits appears to be the secondary metabolites, which include glycoalkaloids, antioxidant chemicals, and vitamins (Saha et al., 2023). Recent reports have shown a strong link between the frequent consumption of phytochemicals and disease prevention (Sharma and Kaushik, 2021). *Ralstonia solanacearum* is known to have a significant impact on economically important crops, such as potato, tomato, tobacco, chili, and eggplant, by causing wilt. The bacterial wilt is most prevalent during the vegetative growth stage (Hussain and Abid, 2011). This disease is one of the major causes of significantly low eggplants yields in Pakistan; therefore, control measures should be adopted to prevent the spread and severity of diseases. In different parts of the world, the causal agent of bacterial wilt is responsible for massive losses in a variety of plants, particularly those belonging to the Solanaceae family (Kongkiattikajorn and Thepa, 2007). It is estimated that *Ralstonia solanacearum* costs the global economy approximately $1 billion annually (Champoiseau et al., 2009). Biochar, a carbon-rich product, is produced from organic feedstock using thermal combustion techniques with limited oxygen (Širić et al., 2023). The International Biochar project claims that biochar is a pyrolysis by-product with a high carbon composition. Biomass is pyrolyzed under anaerobic (oxygen-controlled) conditions to produce biochar. Pyrolysis involves heating a variety of organic wastes to temperatures ranging from 200 to 900°C (Širić et al., 2022). The application of Biochar results in plant development and the reduction of plant diseases (Elad et al., 2011). Biochar improves water retention, enzymatic activity, and cation exchange capacity in soil, reducing salinity stress and promoting the growth of plants and the survival of beneficial microbes (Patel et al., 2017). According to Graber et al. (2010), the population of *Trichoderma* spp. increased in soils receiving biochar compared to control soils. Hu et al. (2014) also documented alterations in the fungal community after applying biochar to the soil, showing a proportion of *Trichoderma* spp. that was 14.5 % greater than in soil that had not been amended with biochar.

*Trichoderma* spp. are beneficial fungi that act as bio-control agents against plant pathogens and promote plant development (da Silva et al., 2016). The fungal symbiosis increases the surface area of roots, leading to increased absorption and subsequently enhancing resistance to plant pathogens (Oskiera et al., 2017). *Trichoderma* also promotes soil preservation by boosting seed germination rates, reducing the effects of abiotic interference, and building a systemic defense that improving soil productivity (Das et al., 2022). Studies have demonstrated the advantages of applying biochar to soil. Patel et al. (2017) use *Trichoderma* as a tool to encourage plant growth and biological control. *Trichoderma* thrives in soil with an abundance of organic materials. The application of *Trichoderma harzianum* in conjunction with organic waste resulted in higher levels of accessible Phosphorus (P) and promoted soil carbon fixation (Khomari et al., 2018). According to Muter et al. (2017), the treatment of biochar together with *Trichoderma* improved the germination of maize seeds and biomass production.

To protect the environment from toxic agrochemicals and crops from phytopathogen-induced damages, it is necessary to devise new disease management strategies. Therefore, this research was conducted to investigate the relationship between biochar and *T. harzianum* while considering the plant response against invading *Ralstonia solanacearum*. Additionally, it would be beneficial to understand the synergistic potential of biochar and BCA on the growth and defense-related biochemical parameters of eggplant.

## 2 Materials and methods

### 2.1 Biochar production

The leaves of *Syzygium cumini* were pyrolyzed at 450°C to create leaf waste biochar (LWB). TLUD (Top-lit Updraft), the portable kiln technique, was used to make biochar with a few modifications (Aftab et al., 2022). The primary burner is a large drum filled with leaf waste. Airflow is controlled by creating holes in the base of the primary burner and using an adapter and chimney. Bricks were used to elevate the burner allow air to enter through the holes. After pyrolysis, tap water was sprayed on the biochar to reduce the temperature, and a sieve was used to powder the biochar.

### 2.2 Soil preparation and experimental plan

The BD (bulk density) of the formalin-sterilized soil was 1.30 g/cm^−3^. The soil substrate was classified as sandy loam, containing 5% clay, 41% silt, and 52.2% sand (>63 mm) (Rasool et al., 2021). Different volumetric concentrations (3% and 6%) of biochar were used as soil amendments either inoculated or uninoculated with *R. solanacearum* in the experimental setup. The experimental setup consisted of the following treatment plan: (a) only soil, (b) 3% leaf waste biochar (LWB), and (c) 6% LWB amended soil substrate for eggplant cultivation. The plants were either inoculated with or without the bio-control agent *Trichoderma harzianum* (+BCA and –BCA, respectively), as well as infected (+RS) with *Ralstonia solanacearum* or remained un-inoculated (–RS). Each treatment had 10 replicates assigned to it in a completely randomized (CRD) experimental design. The characteristics of soil, leaf waste biochar and compost are shown in Table 1.

**Table 1:** Characterization of soil, compost and leaf waste biochar (LWB).

**Parameter**	**Soil**	**LWB**	**Compost**
Nitrogen (%)	0.05	0.74	1.20
Phosphors (%)	2.12	0.69	0.40
Potassium (%)	1.91	0.62	0.55
C/N Ratio	20	68.91	24.16
EC (mS/cm)	0.62	1.77	1.28
Organic matter (%)	0.609	62.86	19.10
pH	8.04	9.25	7.08

### 2.3 Isolation of pathogen and acquisition of *Trichoderma harzianum*

Approximately 10 cm long wilted plant stem segments were chopped into small pieces after being superficially cleaned with 70% ethanol. The cut pieces were then shaken for 5 min at room temperature with 5 sterilized distilled water. Each bacterial suspension was streaked separately onto the bacterial growth medium, ensuring even distribution, and incubated at 28°C for 24 h (Hugh and Leifson, 1953). To get pure cultures, one colony that appeared smooth, circular and dirty white from each bacterial culture isolated from the soil and stem was re-streaked on nutrient agar medium in a sterilized environment. The pathogen inoculum was prepared by stirring bacterial cells in LB broth at 37°C for 48 h (Kim et al., 2016). The *Trichoderma harzianum* culture was provided by the First Fungal Culture Bank of Pakistan (FCBP), Department of Plant Pathology, University of the Punjab (FCBP-SF-1277), w in collaboration with the World Culture Collection Center. Malt extracts agar (MEA) was used for the multiplication of *Trichoderma* spp. (Sunesson et al., 1995).

### 2.4 *Ralstonia solanacearum* inoculum preparation

The inoculum of *R. solanacearum* was prepared by stirring bacterial cells in LB broth at 37°C for 48 h (Kim et al., 2016). The final bacterial culture concentration was adjusted to 108 CFU/mL (OD600 = 0.8) for inoculation. The concentration was adjusted by calculating the optical density using a spectrophotometer.

### 2.5 Application of bio-control agent (*T. harzianum*)

*Trichoderma harzianum* was cultured on MEA media plates at 25°C for 7 days to create a conidial suspension. The conidia were collected from the surface of the plates and then suspended in 0.05% Tween 20 after being cleaned with sterile distilled water. The fungal spore count was increased to 105 spores per mL (Khaledi and Taheri, 2016). The eggplant seeds were surface-sterilized using 50% commercial bleach (3.8% NaOCl) (Davoudpour et al., 2020). The seeds were then submerged in a 10 mL suspension of 105 mL^−1^ spores, agitated with an electric shaker for 0.5 h at room temperature, and allowed to air dry in a laminar flow hood. Methyl cellulose was used to keep the conidia attached to the seed surface. Methylcellulose is typically used as a sticker in bio-control agent formulations at a rate of 2.5% (Larena et al., 2010), and it was mixed with the conidial suspension. Additionally, methylcellulose acts as a shield, protecting the bio-control agent from abrasive physical or chemical treatment. For the soil application of the bio-control agent *Trichoderma* spore suspension, it was applied to already prepared biochar mixed soil pots (Khaledi and Taheri, 2016).

### 2.6 Inoculation of pathogen

The roots of eggplant seedlings were soaked in a bacterial culture for up to 3 min before being planted in pots (Yanti et al., 2017).

### 2.7 Eggplant growth assessment

Agronomic plant physiological variables, such as plant height, root length, above- and below-ground biomass dry weights, were measured 40 days after inoculation. The separated eggplant stems and roots were dried in a 70°C oven until they reached a constant weight (Carter et al., 1994).

### 2.8 Biochemical analysis of eggplant

Total phenolic contents were calculated using the modified Folin-Ciocalteu technique (Singleton and Rossi, 1965). The extracts (1 mL) were mixed with Na_2_CO_3_, the Folin-Ciocalteu reagent (0.5 mL), and the reagent. The absorbance value at 760 nm was determined after 10 min of incubation using a spectrophotometer (Perkin Elmer Lambda-EZ200). Gallic acid equivalent (GAE) was used to express the overall phenolic content of the samples.

The total flavonoid contents of the crude extract were determined using the aluminum chloride (AlCl_3_) colorimetric method (Kaur and Kapoor, 2002). After being homogenized in 3 mL of 80% aqueous ethanol, 250 mg of eggplant leaf tissue was kept at 37°C in the absence of light for 40 min. The supernatant was collected after centrifugation at 10,000 rpm and mixed with 4.3 mL each of 80% aqueous ethanol, 10% aluminum nitrate and 1 M sodium acetate. The absorbance reading was noted at 495 nm following 30 min of dark incubation (Chang et al., 2002). The amount of total flavonoids in the tissue sample was estimated as mg/g of fresh weight of eggplant leaf tissue.

Peroxidase activity was measured by taking 0.5 mL of enzyme extract in a mixture containing 2 mL of phosphate buffer, 1 mL of 0.05 mol L-1 H_2_O_2_ and 1 mL of pyrogallol. Then the mixture was incubated at 25 °C. H_2_SO_4_ (2.5 mol L-1) was added to stop the reaction. The reading was taken at a wavelength of 420 nm against a pyrogallol-removed blank.

### 2.9 Nutrient content determination

To determine the amounts of nitrogen, phosphate, and potassium in eggplant shoots, we used the Kjeldhal technique (Estefan, 2013). The plants were dried and prepared for examination before being powdered. The substances were then digested to determine the amount of N. We digested 0.5 g of the material with 10 mL of H_2_SO_4_ at 420°C for 2 h, using K_2_SO_4_ and CuSO_4_ as catalysts in a 9:1 ratio. To measure the total P and K in the plant material (Estefan, 2013), we used the ICARDA manual wet-digestion method. Spectrophotometers and flame photometers were used to measure the amounts of P and K, respectively.

### 2.10 Disease assessment

Following a modified method of Winsted and Kelman, we recorded disease rating for evaluating disease symptoms and level of infection in plants (Winstead, 1952). The disease evaluation scale is given in Table 2.

**Table 2:** Disease rating scale for *Ralstonia solanacearum* (Kaur and Kapoor, 2002).

**Rating scale**	**Disease percentage**	**Disease response**
0	No plants wilted	I
1	20% plants wilted	HR
2	21–40% plants wilted	R
3	41–60% plants wilted	MR
4	61–80% plants wilted	S
5	More than 80% plants wilted	HS

For the determination of disease incidence, prevalence, and percent disease index, a disease evaluation was done using the following formula (Wheeler, 1969):


$$\begin{array}{c} Percent\;Disease\;Index=\\ \frac{Sum\;of\;all\;rating\;\times 100}{Total\;no.\;of\;observation\;\times \;Maximum\;rating\;grade} \end{array}$$

Disease incidence of bacterial wilt was calculated 40 days after inoculation as percentage of diseased plants in treatment by using the following formula (Awan et al., 2018; Awan and Shoaib, 2019):


$$\begin{array}{c} Disease\;Incidence\;\left(\%\right)=\;\frac{Number\;of\;diseased\;plants}{Total\;no.\;of\;plants}\;\times 100 \end{array}$$

The following formula was used to compute the percentage of protection provided by biochar, where A represents the PDI of untreated control plants and B represents the PDI of treated plants (Attia et al., 2022):


$$\begin{array}{c} Protection\;\left(\%\right)=\;\frac{A-B}{A}\;\times 100 \end{array}$$

### 2.11 Statistical analysis

The data were analyzed using the software Statistix 8.1 to determine the homogeneity of variance. Subsequently, the treatments underwent a three-way analysis of variance (ANOVA), with the factors of analysis being soil composition, *Trichoderma harzianum* and *R. solanacearum*. The means were then compared using Tukey's HSD all-pairwise comparisons test (*P* ≤ 0.05).

## 3 Results

### 3.1 Plant growth-related agronomical parameters

Both the bio-control agent and soil substrate composition had a significant impact on the evaluated agronomic parameters of eggplant under *Ralstonia solanacearum* stress. However, the root and shoot lengths, as well as the dry weights, were strongly influenced by the interaction between soil composition (association with *T. harzianum*) and *R. solanacearum* (Table 3). Generally, the pathogen inoculation had an inhibitory effect on eggplant physiological parameters. However, 3-way (BCA × SC × RS) significant interactive effect was recorded in shoot dry weight. Results of three-way ANOVA are summarized as *P* values in Table 3.

**Table 3:** The level of significance of the relationship between *Trichoderma harzianum* (BCA)*, Ralstonia solanacearum* (RS), and soil substrate composition on eggplant development and physiological parameters was determined by the results of a three-way ANOVA.

**Treatment**	**Shoot length**	**Root length**	**Shoot dry weight**	**Root dry weight**	**N**	**P**	**K**	**Total phenolic**	**Flavonoids**	**Peroxidases**
BCA	^***^	^***^	^***^	^***^	^***^	^***^	^***^	^***^	^***^	^***^
RS	^***^	^***^	^***^	^***^	^***^	^***^	^***^	^***^	^***^	^***^
SC	^***^	^***^	^***^	^***^	^***^	^***^	^***^	^***^	^***^	^***^
BCA × RS	Ns	^***^	^**^	^**^	^***^	^**^	^**^	^***^	^**^	^***^
BCA × SC	^***^	^***^	Ns	^***^	^**^	^**^	^**^	^***^	^***^	^***^
RS × SC	^***^	Ns	Ns	^*^	Ns	Ns	Ns	^**^	Ns	^**^
BCA × RS × SC	^*^	Ns	^*^	Ns	^**^	Ns	Ns	^**^	Ns	^**^

^***^P ≤ 0.001, ^**^P ≤ 0.01, ^*^P ≤ 0.05, and Ns, Non-significant.

#### 3.1.1 Shoot length of eggplants

Plant height was significantly reduced in all treatments inoculated with *R. solanacearum* (Table 3). However, the highest plant shoot length (32.10 cm) was observed in the un-inoculated (–RS) treatment, where 6% LWB soil amendment was used in association with *T. harzianum* treatment. On the other hand, the lowest shoot length (16.50 cm) was recorded in the non-amended soil control group under disease stress (+RS) (Table 4), whereas in the non-amended soil control group under pathogen stress, there was an increase of 52.12% and 72.72% for plants grown in 3% and 6% LWB, respectively. There was an increase of 79.69% in the shoot length of plants grown the in 6% LWB amended treatment, which received both the pathogen and the bio-control agent.

**Table 4:** Effect of leaf waste biochar (LWB) and *Ralstonia solanacearum (RS)* on plant physiological parameters of eggplant cultivated in following soil compositions: only soil (S), soil with 3% leaf waste biochar, and soil with 6% leaf waste biochar associated with and without bio-control agent (+ and –BCA, respectively) either inoculated (+RS) or un-inoculated (–RS).

**Treatments**	**Shoot length (cm)**	**Shoot dry weight (g)**	**Root length (cm)**	**Root dry weight (g)**
S	20.6 ± 0.51^h^	2.24 ± 0.04^ef^	10.15 ± 0.26^e^	0.61 ± 0.005^f^
S + RS	16.5 ± 0.39^i^	1.52 ± 0.09^g^	8.5 ± 0.27^f^	0.51 ± 0.02^g^
S + BCA	24.5 ± 0.316^g^	2.47 ± 0.09^de^	11.6 ± 0.23^cd^	0.71 ± 0.01^def^
S + BCA + RS	22.1 ± 0.245^h^	2.27 ± 0.05^ef^	11.25 ± 0.18^d^	0.66 ± 0.01^ef^
S + 3% LWB	26.56 ± 0.26^ef^	2.49 ± 0.04^de^	12.49 ± 0.27^bc^	0.75 ± 0.01^cd^
S + 3% LWB + RS	25.1 ± 0.4^fg^	2.04 ± 0.09^f^	11.1 ± 0.19^de^	0.65 ± 0.01^ef^
S + 3% LWB+ BCA	28.22 ± 0.68^cd^	3.05 ± 0.06^b^	12.75 ± 0.11^b^	0.80 ± 0.004^cd^
S + 3% LWB + BCA + RS	27.45 ± 0.27^de^	2.67 ± 0.06^cd^	12.57 ± 0.28^bc^	0.75 ± 0.02d^e^
S + 6% LWB	29.95 ± 0.35^b^	2.97 ± 0.06^bc^	14.55 ± 0.22^a^	1.05 ± 0.04^b^
S + 6% LWB + RS	28.5 ± 0.14^bcd^	2.66 ± 0.08^cd^	13.3 ± 0.14^b^	0.85 ± 0.01^c^
S + 6% LWB + BCA	32.10 ± 0.58^a^	3.41 ± 0.06^a^	15.35 ± 0.1^a^	1.20 ± 0.04^a^
S + 6% LWB + BCA + RS	29.65 ± 0.58^bc^	3.18 ± 0.06^ab^	15.15 ± 0.27^a^	1.10 ± 0.016^ab^

All values represent mean ± SE, mean values with top lettering suggesting difference among treatments means at *P* ≤ 0.05.

#### 3.1.2 Root length of eggplants

The highest root length (14.95 and 15.19 cm) was recorded in the treatment where 6% leaf waste biochar was amended in association with *T. harzianum* (S+6%LWB+BCA), whether infected (+RS) or uninfected (–RS) with *R. solanacearum*, respectively (Table 4). In the 3% LWB amended treatment, there was an increase of 35.29% (–BCA+RS) and 47.88% (+BCA+RS) compared to their respective non-amended soil control. However, the lowest root length (8.50 and 10.15 cm) was recorded in soil only potting media (control treatment) in the –RS and +RS treatments, respectively.

#### 3.1.3 Shoot dry weight

The shoot dry weight of eggplants was significantly reduced under bacterial wilt stress. While in 6% LWB, there was an increase of 16.71% and 11.81% in shoot dry weight in comparison with 3% LWB amended treatment association with (+BCA) or without (BCA) bio-control agent *Trichoderma harzianum*, respectively (Table 4). In the presence of *R. solanacearum*, the highest shoot dry weight (1.10 g) was observed in 6% biochar associated with BCA treatment (S+ 6% LWB+ BCA+ RS). On the other hand, the lowest shoot dry weight (1.52 g) was recorded in the non-amended soil control.

#### 3.1.4 Root dry weight

With or without biochar and the bio-control agent (*T. harzianum*), the inoculation of *Ralstonia solanacearum* resulted in a decrease in the root dry weights of eggplant in all treatments (Table 4). The highest root (1.10 and 1.20 g) dry weight was noted in 6% LWB amended soil associated with *T. harzianum* treatment, whether infected (+RS) or un-infected (–RS) with pathogen, which was 60% >3% LWB amended soil associated with *T. harzianum* treatment under pathogen stress (S+ 3% LWB+ BCA+ RS), whereas the lowest root dry weight (0.51 g) was recoded in soil in the absence of LWB and BCA under *R. solanacearum* stress.

### 3.2 Disease assessment

The response of the eggplant to *R. solanacearum* inoculation varied from resistant to highly susceptible with respect to soil composition (Table 5). Plants grown in soil treated with *Ralstonia solanacearum* (S+RS) showed a highly susceptible response to bacterial wilt, with the highest PDI of 88% and 100% disease incidence. The highest plant defense response against the development of bacterial wilt was observed in the treatment containing 6% biochar and *T. harzianum* (S+6% LWB+ RS+ BCA), with minimum (8%) recorded PDI, DS (8.81%) and DI (20%). In association with the bio-control agent, 6LWB provided maximum (90%) protection against bacterial wilt on the eggplants, while it was at 63% in case of plants grown in 3% LWB (Table 5).

**Table 5:** Estimation of disease incidence (DI), disease severity, percent disease index (PDI), percentage protection and plant disease response (PDR) parameters for bacterial wilt of egg-plants cultivated in different soil substrate compositions in association with bio-control agent (BCA) *Trichoderma harzianum*.

**Treatments**	**DI (%)**	**DS (%)**	**PDI**	% Protection^*^	**PDR**
S + RS	100	80.58^a^	88	–	HS
S + BCA + RS	80	59.14^ab^	48	45	S
S + 3% LWB + RS	80	55.52^ab^	40	54	S
S + 3% LWB + RS + BCA	60	27.66^ab^	32	63	MR
S + 6% LWB + RS	40	18.08^b^	16	81	MR
S + 6% LWB + RS + BCA	20	8.81^b^	8	90	R

^*^Plant protection from *Ralstonia solanacearum* of biochar amended treatments in comparison to soil only treatment. Superscript letters (a, b, and ab) indicate significant differences among values with in a column.

### 3.3 Nutritional content of eggplants

There was a significant 3-way (SC × BCA × RS) interaction between soil composition with the bio-control agent, and *R. solanacearum* on the N, P, and K contents of eggplants (Table 3). Growing eggplants in 6%LWB+BCA amended soil with or without *R. solanacearum* stress had a positive impact on the nutritional content of the plants.

**Figure 1:**
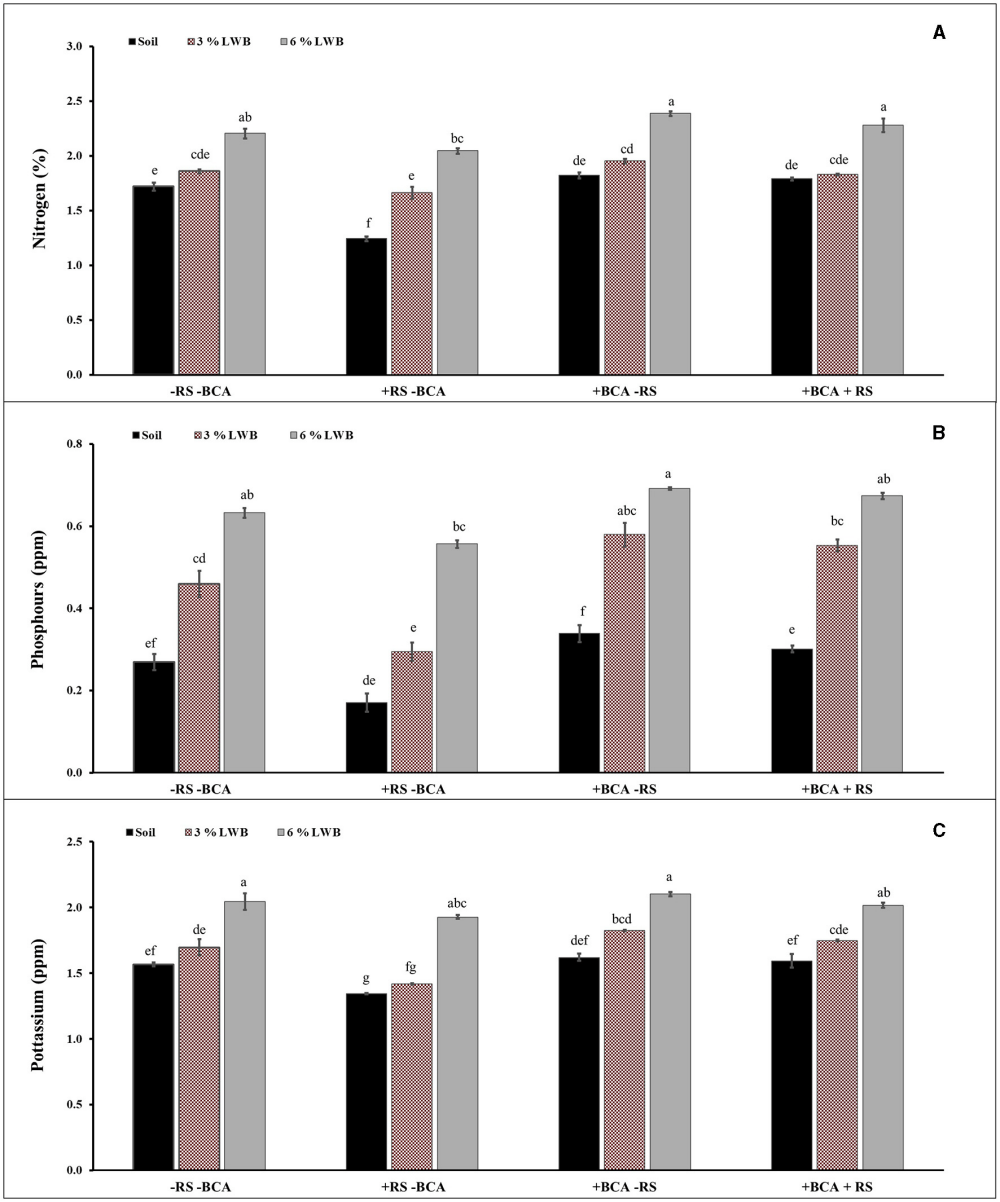
Effect of leaf waste biochar and *Ralstonia solanacearum* on nitrogen **(A)**, phosphorus **(B)**, and potassium **(C)** content of eggplant cultivated in the following soil compositions: only soil, soil with 3% LWB, and soil with 6% LWB associated with and without bio-control agent (+ and –BCA, respectively), whether infected (+RS) or non-infected (–RS). All values represent mean ±SE, and bars with top lettering suggest the difference among treatments at the *P* ≤ 0.05.

The effect of soil composition in association with the bio-control agent and *R. solanacearum* was significant (*P* ≤ 0.01) for nitrogen (N) content of eggplants (Table 3). Figure 1A shows a higher percentage of N content in 6% the leaf waste biochar treatment, with or without the bio-control agent. Highest nitrogen (2.28 and 2.39 %) content was observed in the 6% biochar amendment along with BCA treatment, whether infected (+RS) or uninfected (-RS), respectively (Figure 1A). Among all biochar amended treatments associated with the bio-control agent, there was an increase of 24.59 and 22.56% of plant N content in the 6% LWB containing soil as compared to the 3% LWB amended soil, whether infected (+RS) or uninfected (–RS), respectively. The lowest nitrogen content (1.24%) was recorded in the non-amended soil control under pathogen stress.

A significant (*P* ≤ 0.01) interaction effect of BCA × RS and BCA × SC was observed in the case of phosphorus content (ppm) (Table 3). The highest significant Phosphorus content was observed in plant grown in soil amended 6% LWB in association with bio-control agent either infected or uninfected with *R. solanacearum* (0.67 and 0.69 ppm), respectively (Figure 1B). Whereas in remaining (3% LWB) biochar amended treatments inoculated with *R. solanacearum*, both with or without bio-control agent the phosphorus content was significantly reduced 21.81% and 18.96%, respectively, in comparison with plants grown in 6% LWB. However, the lowest (0.17 ppm) P content was observed in *R. solanacearum* infected plants raised in non-amended soil only treatment. Under the pathogen stress, there was a significant increase of 76.47% of phosphorus contents in plant grown in soil amended with 6% LWB in the presence of bio-control agent, when compared with non-amended soil control.

Bacterial wilt infection in eggplant had a significant impact on reducing the K content (Table 3). Eggplants in soil amended with 6% LWB associated with biocontrol agent had shown the highest (2.06 ppm) K contents. The association of biocontrol agent *T. harzianum* also significantly influenced the potassium content in eggplants. The lowest (1.34 ppm) K content was recorded in non-amended soil control under *R. solanacearum* stress (Figure 1C). The K contents among all the treatments were recorded from lowest to highest in the following order: soil<3% LWB<6% LWB, either inoculated (+RS) or non-inoculated (–RS).

### 3.4 Biochemical analysis

**Figure 2:**
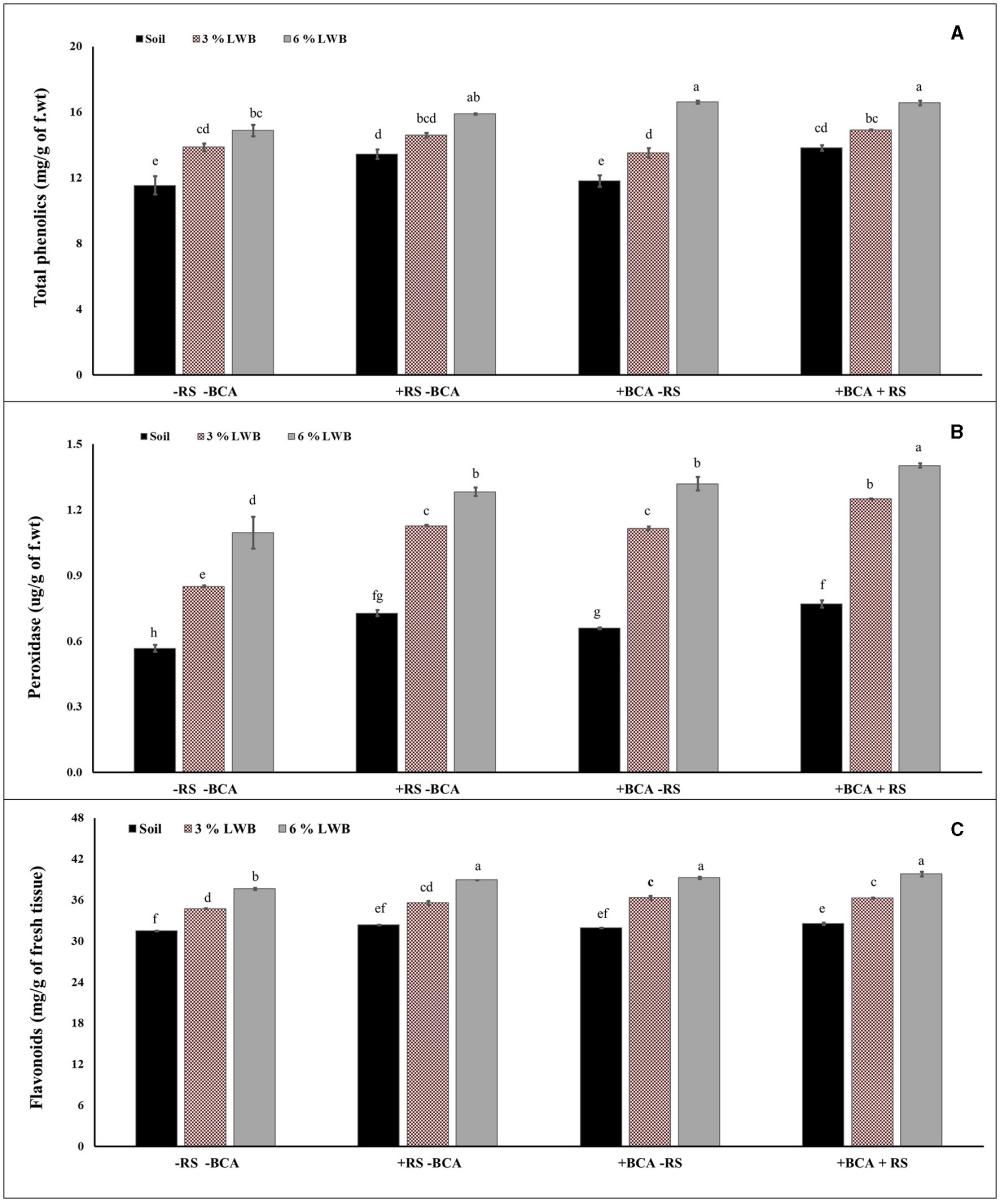
Effect of leaf waste biochar and *Ralstonia solanacearum* on total phenolics **(A)**, peroxidases **(B)**, and flavonoids **(C)** content of eggplant cultivated in following soil compositions: soil, soil with 3% LWB, and soil with 6% LWB associated with and without the bio-control agent (+ and –BCA, respectively), whether infected (+RS) or uninfected (–RS). All values represent mean ±SE, and bars with top lettering suggest the difference among treatments at *P* ≤ 0.05.

The association of a bio-control agent with biochar had a significant impact on the biochemical content in eggplants. A three-way strong interactive effect was observed between the bio-control agent, different soil compositions and *R. solanacearum*, which influenced the production of biochemicals in eggplants (Table 3). The phenolic content increased (12.73%) significantly in the 6% biochar-amended treatment compared to plants in the 3%LWB co-inoculated (BCA+RS) (Figure 2A) treatment. In plants grown in the 6% LWB without BCA association (6% LWB-BCA), there was an increase of 7.19% (in comparison with 3%LWB-BCA) under disease stress (+RS). *Ralstonia solanacearum* significantly increases the total phenolic content of eggplants, with a maximum of 18.51% in plants grown under the 6% LWB amended treatment, compared to the non-amended soil only treatment (Figure 2A).

The combined effect of *T. harzianum* and biochar significantly increased the production of peroxidase. Among the biochar amended treatments, the highest (1.42 ug/g of f.wt) peroxidase activity was observed in eggplants grown in 6% leaf waste biochar amended co-inoculated (BCA + RS) treatment, whereas the lowest (0.57 ug/g of f.wt) peroxidase activity was recorded for non-amended soil control treatment in the absence of pathogesn (Figure 2B). However, there was a significant difference in peroxidase production between the 3% and 6% biochar amended treatments and soil composition in the presence and absence of *R. solanacearum* and *T. harzianum*.

The highest concentration of flavonoids was recorded in plants grown in the 6% LWB treatment in association with *T. harzianum*, whether they were inoculated (+RS) or un-inoculated (–RS) (39.81 and 39.25 mg/g of fresh tissue, respectively) (Figure 2C). In the absence of biochar and the bio-control agent, flavonoids' concentrations were found to be the lowest (31.49 mg/g of fresh tissue) in soil only treatment along with pathogens. The flavonoid content increased significantly in co-inoculated plants (+RS+BCA) with the increase in biochar concentration, that is, 9.72% increase was recorded in plants grown in the 6% LWB amended soil substrate compared to the respective 3%LWB counterpart.

## 4 Discussion

Plant diseases are primarily responsible for the decline in both the quality and quantity of agricultural products (Savary et al., 2019). In order to move toward a sustainable agricultural system in the 21^st^ century, it is essential to thoroughly assess different crop production strategies. Sustainable agriculture aims to meet current needs without jeopardizing the ability of future generations to satisfy their own needs. By understanding ecosystem services, sustainable agriculture aim to create a more environmentally pleasant world. Due to plant pathogens induced reduction in the quantity and quality of agricultural products, agri-business is severely hampered (Savary et al., 2012). In a recent study, Širić et al. (2022) found that the cauliflower plants benefited from both PGPR and biochar, in terms of growth improvement and yield enhancement. Microorganisms in the PGPR-based bio-fertilizer adhere to the biochar particles. The biochar particles serve as a host, giving the microorganisms a carbon niche for their better growth and development (Tao et al., 2018).

Despite biochar being used for a long time to improve soil quality, researchers have recently developed a specific interest in its potential for enhancing plant growth and carbon sequestration (Lehmann et al., 2011). The application of biochar can change how plants react to disease stress (Graber et al., 2014) in addition to having an effect on crop yield (Kloss et al., 2014). In the past, biochar has been shown to reduce bacterial and fungal soil-borne diseases in tomato plants. However, no research has been conducted to evaluate the effect of biochar on bacterial wilt in eggplants. To our knowledge, the findings presented are the first to demonstrate the impact of biochar (with or without a bio-control agent) on the development of a soil-borne pathogen (*R. solanacearum*) in eggplants.

It is widely known that bio-control agents, such as *Bacillus subtilis* (Shoaib et al., 2019; Attia et al., 2020), *Trichoderma* spp. (Freeman et al., 2004), *Penicillium axalicum* (De Cal et al., 2008), and others can induce resistance in plants against various pathogens, including *R. solanacearum, A. solani, Colletotrichum acutatum*, and *Botrytis cinerea*. In a recent study by Rasool et al. (2021), it was found that the application of biochar along with PGPR (plant growth promoting rhizobacteria) had a reducing effect on the incidence and severity of *solani*. They attributed the decrease in disease development in tomatoes to the up-regulation of defense-related gene expression.

*Trichoderma harzianum* is a free-living organism that exhibits antagonistic action against several phyto-pathogenic fungi and bacteria on roots and in plant tissues (Rajendiran et al., 2010). Globally, *T. harzianum* is one of the most extensively researched and utilized bio-control agents for plant diseases. According to Graber et al. (2010), the population of *Trichoderma* spp. increased in soils treated with biochar compared to control soils. Hu et al. (2014) also observed changes in the fungal community after the application of biochar to the soil, with the proportion of *Trichoderma* being 14.5% higher in soils with biochar compared to those without. The connection between *T. harzianum* and root leads to an increase in the area of root absorption which in turn enhances nutrient absorption and then increases the pathogen resistance factor (Oskiera et al., 2017). Similarly, the present results indicated that the biochar along with *T. harzianum* improves t plant's ability to cope with *R. solanacearum*. *Trichoderma* associated with higher (6%) concentration of biochar had a strong impact on eggplants compared to plants grown in 3% leaf waste biochar amended soil. The advantages of applying biochar to soil along with the use of *Trichoderma* as a tool to promote plant growth and biological control have been documented (Patel et al., 2017). The application of *Trichoderma harzianum* along with organic waste resulted in levels of accessible P and promoted soil carbon fixation (Khomari et al., 2018). According to Muter et al. (2017), the treatment of biochar together with *Trichoderma* improved the germination of maize seeds and produced higher plants. Biochar in combination with vermi-compost known to have stimulating effect on plant growth as well as on the fungal and bacterial populations in the soil (Wang et al., 2021).

The highest concentration of nitrogen in plants was attributed to increased availability and reduced leaching of mineral nutrients. According to Chan et al. (2007), the use of biochar together with mineral fertilizers had a positive effect on nitrogen uptake. Prior research by Doan et al. (2015) reported beneficial effects of increasing nitrogen availability on the development and production of dry matter in maize plants. Several studies have demonstrated that the application of organic fertilizers, such as biochar and compost, along with mineral fertilizers stimulated plant growth and improved the efficiency of the fertilizer (Alburquerque et al., 2013). Previous studies had also shown the combined use of spent mushroom substrate biochar and PGPR improved the nutrients contents of cauliflower (Širić et al., 2022).

According to a study by Agegnehu et al. (2016), biochar and compost application serve as a source of transferable and easily accessible phosphorus. Due to phosphorus precipitating as dicalcium phosphate, the calcareous soils are phosphorus deficient (Abbas et al., 2010). However, soil biochar amendments enhance phosphorus availability, thus, resulting into improved plant growth (Nigussie et al., 2012). Similar to this, Steiner et al. (2008) has shown that the addition of compost and biochar enhanced crop stand, growth, and yield, which resulted in increased plant absorption of potash. Increased nitrogen and phosphorus availability may be directly or indirectly responsible for the rising potassium content in plants and soil. It can be believed that the increased nutritional concentration in biochar results from the saturation of nutrients during the pyrolysis of biomass.

In addition to harming the environment and human health, the over use of agricultural pesticides also speed-up the process of evolution of pathogenic strains, making them immune to commonly employed disease management techniques (Schmitz et al., 2014). The highest amounts of defense-related bio-chemicals were found in eggplants grown in soil that contained both biochar and *Trichoderma*. Phenolics in plants function by boosting the level of defense related proteins, while inducing structural alterations like lignification of the cell wall, as well as reduction in reactive oxygen species induced stress (Ozdal et al., 2013; Kaur et al., 2017). According to reports, tomato plant defense against early blight is boosted by the formation of phenolics and antioxidants (Awan et al., 2018). According to the present findings, *R. solanacearum* infection on eggplants in the biochar soil amendment (with and without BCA) also considerably influenced the levels of catalase and peroxidase synthesis, which in turn helped to mitigate the stress by bacterial infection (Attia et al., 2022). Biochar and PGPR are known to interact with crop plants biochemical responses toward biotic and abiotic stresses. Lalay et al. (2022) studied the combined application of biochar and *Pseudomonas* sp. influenced the concentrations of anthocyanins, carotenoids, chlorophyll pigments and catalases in *Brassica napus* L.

The results of this study have greatly expanded the understanding of how biochar contributes to the development of disease resistance and the transformation of waste products into carbon-rich soil amendments. Organic amendments in association with bio-control agent plausibly increased the soil's organic carbon contents and eventually rendered the soil structure permeable. Biochar addition plausibly influenced the roots to grow deeper into the soil to acquire more nutrients. These findings may also be utilized to create organic materials to replace traditional plant protection practices, for preventing bacterial wilt in eggplants.

## 5 Conclusions

Biochar-based organic soil amendments can strengthen plant defense and manage emerging biotic threats. Eggplant's reaction to the invasive *R. solanacearum* was affected by the presence of biochar in soil substrate. The comparison revealed that *T. harzianum* alone and in combination with 6% leaf waste biochar was the most efficient in suppressing *R. solanacearum*. Using biochar with association of *T. harzianum* increased microbial activity in the soil, which activated plant growth and inhibited further disease progression. The physico-chemical properties of biochar might be influenced by the raw material and temperature used for pyrolysis, which in turn impact how plants will react to phyto-pathogens (biochemically and physiologically). We anticipate greater interest in biochar in the near future from the standpoint of the agricultural industry and an effective waste management plan. To understand the mechanism behind induced resistance, future studies should focus at the molecular plant response to the biochar application. There is also need to standardize the effective doze for various biochars classified on the basis of feedstock to extract the full benefits under field conditions.

## Data availability statement

The original contributions presented in the study are included in the article/supplementary material, further inquiries can be directed to the corresponding author.

## Author contributions

CA: Writing—review & editing, Writing—original draft, Software, Methodology, Investigation, Formal analysis, Data curation. AA: Writing—review & editing, Visualization, Supervision, Conceptualization. MH: Writing—review & editing, Visualization, Validation, Supervision, Conceptualization. MA: Writing—review & editing, Methodology, Investigation, Formal analysis, Data curation. AH: Writing—review & editing, Resources, Project administration, Funding acquisition. GA-Q: Writing—review & editing, Resources, Project administration, Funding acquisition. EA: Writing—review & editing, Resources, Project administration, Funding acquisition.
